# Sulfuric Acid-Catalyzed Dehydratization of Carbohydrates for the Production of Adhesive Precursors

**DOI:** 10.1021/acsomega.1c02075

**Published:** 2021-06-15

**Authors:** Wilfried Sailer-Kronlachner, Catherine Thoma, Stefan Böhmdorfer, Markus Bacher, Johannes Konnerth, Thomas Rosenau, Antje Potthast, Pia Solt, Hendrikus W. G. van Herwijnen

**Affiliations:** † Wood K plus—Competence Center of Wood Composites and Wood Chemistry, Kompetenzzentrum Holz GmbH, Altenberger Str. 69, A-4040 Linz, Austria; ‡ Institute of Wood Technology and Renewable Materials, Department of Material Science and Process Engineering University of Natural Resources and Life Sciences, Vienna (BOKU), Konrad-Lorenz Str. 24, A-3430 Tulln, Austria; § Institute of Chemistry of Renewable Resources, Department of Chemistry, University of Natural Resources and Life Sciences, Vienna (BOKU), Konrad Lorenz-Straße 24/I, A-3430 Tulln, Austria

## Abstract

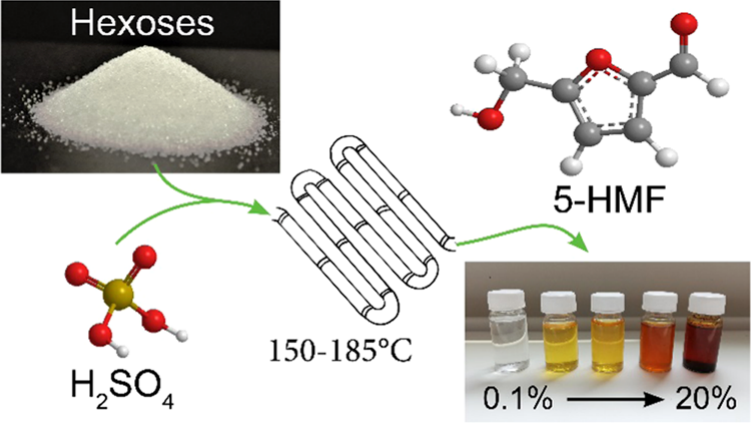

Carbohydrates and hexose-derived 5-hydroxymethylfurfural (5-HMF) are platform chemicals for the synthesis of sustainable binders. New, greener approaches aim at the development of production systems, which minimize process steps and avoid organic solvents or other auxiliaries that could interfere with subsequent resin synthesis. In our work, carbohydrate solutions rich in 5-hydroxymethylfurfural (5-HMF) were produced using a continuous-flow microreactor and diluted H_2_SO_4_ as the catalyst. After optimization of the process conditions (temperature, reaction time, catalyst content), a 5-HMF yield of 49% was obtained at a low reaction time of 0.6 min and a catalyst concentration of 1% at 175 °C and 17 bar pressure. Extensive rehydration of the product was avoided by efficient immediate cooling of the reaction solution. The stability of the reaction system was improved by increasing the inner diameter of the capillary in the flow reactor to 2 mm. Advantageously, the obtained reaction mixtures are used directly as precursors in the development of sustainable binder systems, without the need of additional purification, filtration, or extraction steps.

## Introduction

Today’s chemical industry still strongly relies on oil and other fossil resources as the main source of bulk chemicals and energy. Rising demands and diminishing fossil resources along with rising awareness of environmental problems drive the search for more sustainable alternatives. The interest in fuels and chemicals derived from renewables is therefore growing fast and a lot of R&D is done to convert biomass into valuable products. Biomass is the only widely available carbon source besides oil, gas and coal, and 75% of the available biomass are carbohydrates, such as starch, cellulose, or hemicelluloses.^1^

The conversion of these carbohydrates into valuable chemicals, e.g., furanic compounds such as 5-hydroxymethylfurfural (5-HMF), has therefore huge industrial potential. 5-HMF is considered a key platform chemical since it can be converted into a variety of other valuable compounds. It has been called a “sleeping giant” along with furandicarboxylic acid (FDCA), a compound that can be derived directly from 5-HMF and may be a renewable alternative for terephthalic acid in polyester or polyamide production.^2^

**Figure 1:**
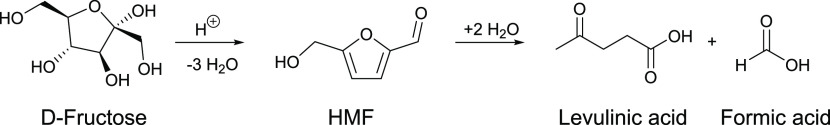
Conversion of fructose to 5-HMF and rehydration to the byproducts levulinic acid and formic acid via side reaction.

Extensive literature on the production of 5-HMF is available, including good overviews of synthesis procedures, solvent systems, and proposed reaction mechanisms (Van Putten et al.^3^, Yu and Tsang^4^ and Hu et al.^5^). Recently, we have added an outline of the challenging development of industrial 5-HMF production processes.^6^ One of the main challenges in 5-HMF production is the formation of side products. In general, hexoses are dehydrated by acid catalysis to form 5-HMF. 5-HMF is easily rehydrated to levulinic and formic acid, on the one hand, and also polymerizes, on the other hand, thereby forming complex, black-colored residues called humins.^7^Figure 1 depicts the conversion scheme of fructose to 5-HMF as well as the common side products levulinic acid and formic acid.

**Figure 2:**
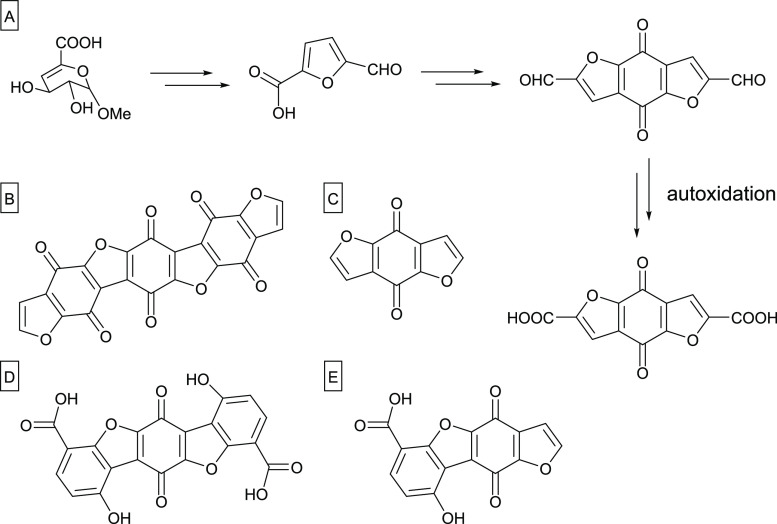
(A) Chromophore formation from hexenuronic acids. (B–E) Isolated chromophore structures in the black humin material; redrawn according to Rosenau et al.^8^

**Figure 3:**
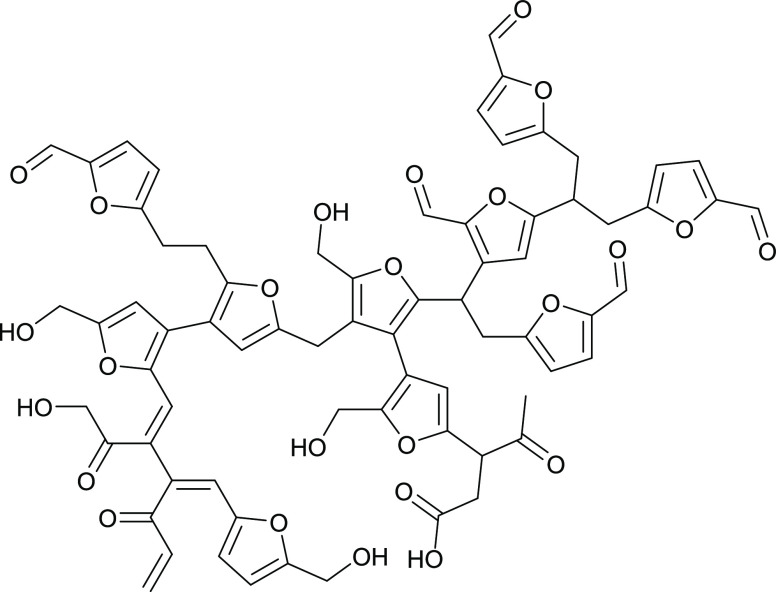
Carbohydrate-derived humin structure, redrawn according to Van Zandvoort et al.^9^

The structure of humins has not been solved completely. Rosenau et al.^8^ presented convincing evidence for a ladder-like structure in their work on chromophores from hexeneuronic acids. They showed that furanic compounds, such as 5-formylfuran-2-carboxylic acid or furan-2-carboxylic acid, form very potent ladder-type chromophores that lead to a coloration of solutions even in minute concentrations of 1 nM. They isolated five of these compounds and proved the formation mechanism by means of ^13^C-isotopic labeling reactions. Diels–Alder reactions are a possible part of the reaction pathway to chromophore formation. In the meantime, it has been established that similar benzoquinone-furanoid chromophores make up 50–70% of the humins mass formed from carbohydrate biomass, depending on the conditions (unpublished results). Van Zandvoort et al.^9^ worked on the formation, molecular structure, and morphology of humins derived directly from sugars like glucose, fructose, or xylose by acidic dehydration. They proposed that humins derived from glucose have a chain-like furanic structure formed according to a dehydration pathway with nucleophilic attacks of the aldehyde moiety in the β-position of the furanic rings and aldol condensations with rehydration products, such as 2,5-dioxo-6-hydroxyhexanal. Figure 2 shows the chromophore structures in humins isolated by Rosenau et al., and Figure 3 displays a structure of glucose-derived humins proposed by Van Zandvoort et al. Humin and chromophore formation was also observed in the present work, leading to dark brown to black reaction solutions, and the minimization of these byproducts was thus an important issue with regard to optimization of reaction conditions.

One of the potential direct applications of 5-HMF is its usage as cross-linker in binder systems.^10^ Initial approaches toward carbohydrate-based adhesives, hypothesizing about in situ 5-HMF formation and its use as a cross-linker, date back to 1926.^11^ Pizzi^12^ defined the use of carbohydrate degradation products as one of three main ways to utilize carbohydrates in wood adhesive systems, the other two being modification of existing adhesive systems and the direct use of carbohydrates as an adhesive. For a more detailed overview of the topic of 5-HMF in binder production, the reader is referred to a recent review by our group.^13^ In binder applications, humins produced during the reaction do not need to be removed as long as they stay in solution, which is an important advantage with regard to overall yield and process simplicity because of the avoidance of separation/purification steps. Recently, Sangregorio et al.^14^ published a paper on humin utilization as resin for wood modification and property improvement. They demonstrated that humins can be valorized as resin in composite materials with good water resistance and tensile shear strength. Of the three main side products of the reaction (formic acid, levulinic acid, and humins), humins are the side product that are believed to be incorporated in the final adhesive network, so enhanced water resistance and a contribution to the adhesive strength can be effects of humins in the final adhesive system.

Our goal is the development of new adhesive systems that have a lower environmental impact as the currently used standard urea-formaldehyde-based adhesives, but still meet the quality standards and fulfill all of the requirements of a modern industry. The produced 5-HMF-rich carbohydrate solutions were to be used directly for binder production. Therefore, no complicated separation or purification steps would be needed. In the production, some criteria needed to be met. To avoid those removal, separation or cleanup steps, a homogeneous catalyst that could remain in the reaction solution was selected. Only solvents considered as “green”^15^ and not interfering with later resin synthesis were of interest, excluding a wide variety of organic solvents and biphasic reaction systems, such as the one used by Roman-Leshkov et al.^16^ who tested a two-phase reactor system with various organic solvents to achieve high yields of up to 73%. Reaction temperature and time are crucial parameters as well, both promoting byproduct (humin) formation—therefore, also heating and cooling times must be as rapid and controllable as possible. Consequently, only very small quantities of reaction solution have been used in most of the published work. These can, in fact, be heated and cooled very efficiently, but are not easily up-scalable in batch processes, let alone compatible with large-scale production. We therefore turned our interest to microreactors, which allow a very good control of these reaction parameters due to the high surface-to-volume ratio of the reagents. Microreactor arrays have a very good upscalability potential, since no variations in batch size need to be considered and the required product quantities can be acquired simply by longer runtimes or by running several reactors in a parallel system.^17^ Several publications on the production of 5-HMF in such reaction systems can be found. Tuercke et al.^18^ achieved yields of 54% in a HCl-catalyzed reaction system, which could even be improved by the addition of a co-solvent, while Muranaka et al.^19^ used a segmented-flow microreactor with saline phosphate buffer as a catalyst and 2-sec-butyl phenol as a co-solvent to achieve yields of up to 80%. Therefore, a microreaction system for the conversion was chosen in this work, utilizing H_2_SO_4_ as the homogeneous catalyst, avoiding corrosive HCl.

The aim of this study was to establish a stable, scalable and green reaction system for the production of 5-HMF-rich carbohydrate solutions to be used directly in binder development without additional purification or separation steps. The addition of organic solvents or heterogeneous catalysts was to be avoided. The influence of the reaction parameters temperature, reaction time, and catalyst content was investigated in detail to maximize the 5-HMF yield. Solvent and catalyst were chosen according to green chemistry standards as well as requirements for industrial production.

## Materials and Methods

Fructose (crystalline, 99.5%) and glucose (powder) were supplied by Cargill, Inc. in high purity. H_2_SO_4_ (96%, Rotipuran, p.a., ISO) was purchased from Carl Roth GmbH + Co. KG and diluted to the desired concentration with deionized H_2_O.

### Batch Experiments

For the preliminary batch experiments, a standard glass apparatus as well as a pressurized steel tank reactor PARR 4842 with a total vessel volume of 0.1 L were used. The PARR reactor was equipped with a heating jacket, a gas inlet, a safety valve, and a sample valve. The conversion of fructose to 5-HMF was performed under ambient atmosphere. The reaction solutions were heated to the desired temperature (120–160 °C) and kept isothermal for the desired reaction time. After the removal of the heating jacket, the tank was cooled in an ice bath. Typically, 40 mL of solution was used in the experiments. The solution was stirred with a propeller stirrer, and the speed of rotation and the temperatures were set from control panel.

**Figure 4:**
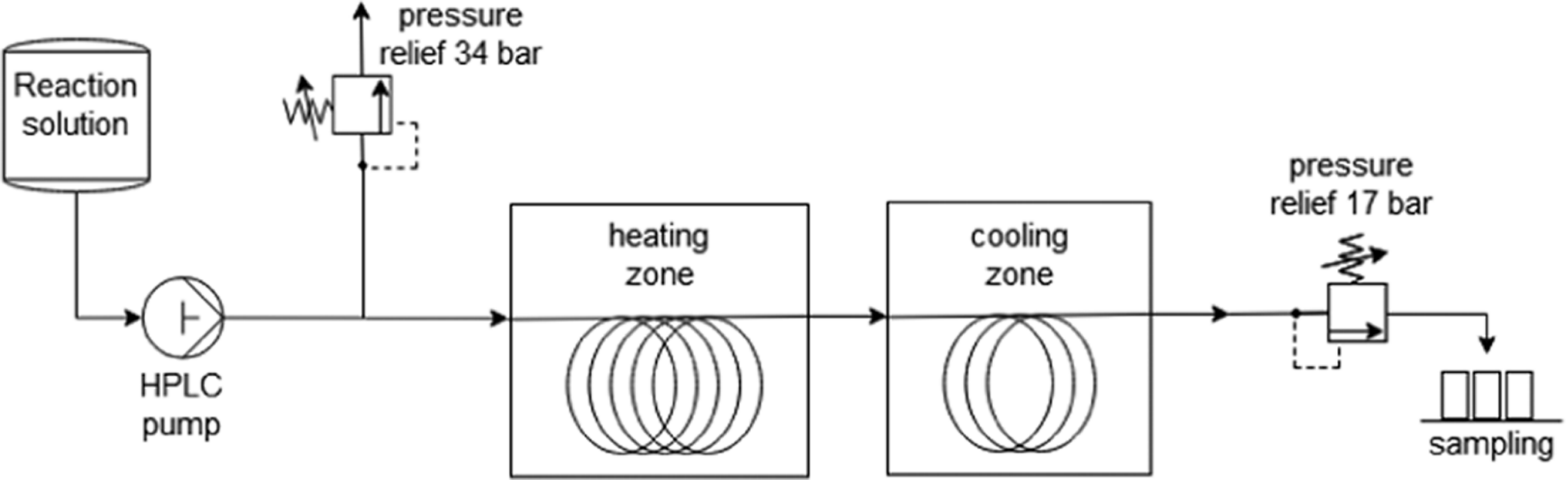
Microreactor setup.

### Microreactor Experiments

The microreactor was built using a steel capillary with an inner diameter of 1 mm and an outer diameter of 1.59 mm (1/16 inch). A Knauer 100 high-performance liquid chromatography (HPLC) pump with a maximum flow rate of 10 mL/min and a ceramic pump head was used to transport the liquid reaction phase, typically consisting of acidic fructose solution, through the heating zone where the conversion to 5-HMF took place. Heating was accomplished by placing the coiled steel capillary in an oil bath, which was kept at the desired reaction temperature (between 120 and 190 °C). After the heating zone, the reaction solution was rapidly cooled by running the capillary through an ice bath. The residence time in the heating zone equals the reaction time and was set by adjusting the flow velocity. Typical reaction times were in the range of 0.5–2.5 min. The overall length of the reactor was 840 cm with a heated reaction zone length of 625 cm leading to an overall volume of the reactor of 6.6 mL with 5 mL in the reaction zone. A safety valve after the HPLC pump and a backpressure regulator (17 bar) after the cooling zone guaranteed a stable flow throughout the system without degassing of the reaction solvent. (Figure 4) The starting fructose concentration was reduced to 5% in comparison to the batch experiments. It was quickly established that higher fructose concentrations bear a higher risk of clogging in the used tubes and valves. Therefore, the optimization was done at a lower fructose content, which was then raised to 10% later on in the mesoreactor system.

### Mesoreactor Experiments

The mesoreactor was built using a steel capillary with an inner diameter of 2 mm, an outer diameter of 1/8″, a reactor length of 320 cm, and a reaction zone length of 160 cm. This results in a reaction zone volume of 5 mL. Besides that, the mesoreactor setup was identical to the microreactor setup.

### Analysis of Reaction Solutions

All reaction solutions were analyzed directly by ^1^H NMR spectroscopy for the determination of the 5-HMF concentration. All ^1^H NMR spectra were acquired at room temperature on a Bruker Avance II 400 instrument (resonance frequency, 400.13 MHz for ^1^H) equipped with a 5 mm liquid N_2_ cooled probe head (Prodigy) with *z*-gradients. For the measurements, 500 μL of the sample solution was mixed with 30 μL of 0.34 mM NaOAc as internal standard and 100 μL of D_2_O (99.8% D, Euriso-top, Saint-Aubin, France). A relaxation delay of 2 s was used.

### Production and Evaluation of Adhesives

The produced 5-HMF solutions are concentrated to a desired 5-HMF content and then directly used in resin synthesis. In a typical procedure, fructose and an amine, e.g., hexamethylenediamine (HMDA), are added to the precursor solution and heated at 60 °C for 20 min. The resulting resin is cooled to room temperature and a second portion of amine is added. The bond strength development for these resins is tested by measuring the tensile shear strength of two beech lamellas glued together at a 120 °C press temperature and varying press times. A more detailed description of the measurement method was published by Solt et al.^20^ All tests were conducted following standard procedures.^21^

## Results and Discussion

### Selection of Starting Material, Catalyst and Solvent

As the conversion of fructose to 5-HMF is faster and more efficient than the conversion of glucose,^3^ which was also confirmed in first-batch experiments, fructose was chosen as the starting material for all conversion experiments.

Water was chosen as reaction solvent for several reasons. Both 5-HMF and fructose are water-soluble and water is the solvent used in state-of-the-art wood adhesives, such as urea-formaldehyde resins. If particle boards are produced with a resin system, water vapor also mediates temperature transfer in the pressing step resulting in temperature-induced hardening of the resin. Other solvents often interfere with the polymerization reactions of the resins and could lead to the formation of other side products, e.g. alcohols can form HMF ethers and (hemi)acetals.^22^ Ionic liquids and deep eutectic solvents have also been used as solvents for the production of 5-HMF. Good yields in the range of 70–78% have been reported by Marullo et al.^23−25^ at remarkably low reaction temperatures of 50–60 °C. They also utilized ultrasound irradiation to improve the process. However, the use of high-boiling or hazardous solvents requires additional separation steps, which would increase energy consumption and raise the overall process costs. Also, the cost of the solvent itself must be considered. Although higher HMF yield and selectivity can be achieved by the use of alternative solvents or biphasic reaction systems,^3^ the above-mentioned reasons advocated for water as the reaction solvent. Although byproduct formation is generally considered as one of the main problems related with the use of water in this reaction system, this problem does not apply to the binder application as these byproducts do not need to be separated and might even have positive effects in the binder, as discussed in the Introduction section.

H_2_SO_4_ was chosen as the acidic catalyst for the reaction. Heterogeneous catalysts were ruled out because additional filtration or separation steps would interfere with the planned direct use of the reaction solution. The decisive factor for the use of H_2_SO_4_ in comparison to a HCl-based reaction system—although slightly higher 5-HMF yields were obtained with HCl in the past^3^—was that H_2_SO_4_ is less corrosive toward steel at higher temperatures than HCl, due to the formation of passivation layers.^26^ Therefore, the long-term stability of a steel capillary system or steel tank reactor is higher if H_2_SO_4_ is used. In addition, the use of chlorine during the syntheses could lead to adsorbable organic halogens (AOX) emissions (e.g., chlorophenols) upon recycling or incineration of the glued products after use.^27^

### Batch Experiments

**Figure 5:**
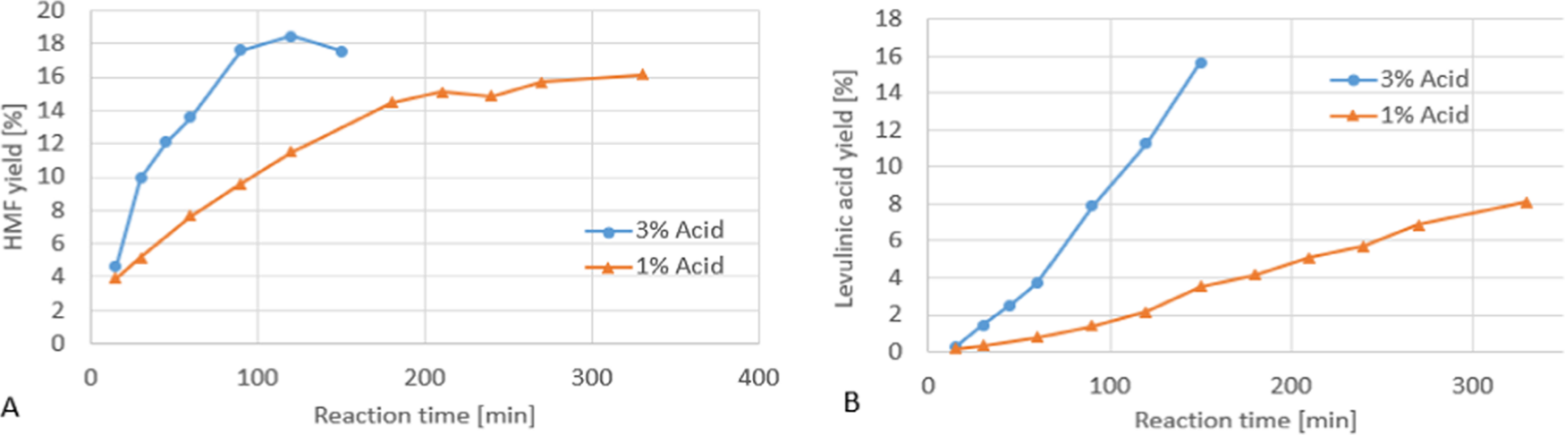
Typical batch conversion of 10% fructose solution in aqueous medium comprising 1 or 3% (v/v) H_2_SO_4_ as the catalyst at reflux temperature. (A) yield of 5-HMF; (B) yield of levulinic acid.

First experiments for the selection of the starting material were conducted in a simple batch setup in a glass flask. Fructose or glucose was dissolved in 1% (v/v) H_2_SO_4_ to create a 10% (w/w) solution and heated to reflux temperature. Samples were taken after the respective reaction times. While the reaction with fructose yielded 16% 5-HMF after 5 h, the conversion of glucose resulted only in a yield of 0.26% 5-HMF. The reaction was repeated with 3% (v/v) H_2_SO_4_, which led to an 18% 5-HMF yield from fructose, and also to significantly more byproduct formation, which is exemplified by the levulinic acid yield in Figure 5.

That led to the conclusion that more 5-HMF was produced in a shorter time frame when 3% H_2_SO_4_ was used, but 5-HMF decomposition into levulinic acid and formic acid by rehydration was promoted. Also, a lower yield was obtained after 150 min than after 120 min, arguing against extended reaction times. The reaction did show a maximum yield of 18% 5-HMF in this particular setting due to the decomposition or polymerization of the main product, which is also the reason for the lower reaction yield after 120 min when 3% H_2_SO_4_ was used. It should be noted that in the case of 3% H_2_SO_4_, a black, carbonaceous material started to form at the interface region of the reaction solution, the glass flask, and the gas phase already early during the reaction. This shows, together with the higher yields of levulinic acid, that more HMF is decomposed or polymerized.

The second-batch reaction system tested was a pressure reactor. Pressurization allowed higher reaction temperatures than possible in an ambient-pressure system. Different reaction conditions were tested with a design of experiment (DoE) approach, including different reaction times, temperatures, fructose contents, and acid contents. The best result obtained was 38% 5-HMF yield (with 11% of levulinic acid side product) starting from a 5% fructose solution in 1% H_2_SO_4_ at 140 °C and 15 min reaction time. Reactions with 3% catalyst content were conducted that lead to mainly humin and side-product formation because the heating and cooling times of the reaction system were too long and faster heating could not be realized.

From these experiments, it was noted that a precise regulation of the pressure reactor’s heating and cooling system is needed, evident from a poor reproducibility of the results. This is important since the large part of 5-HMF is formed during a relatively short time. Thus, if yields are inconsistent even on a lab-scale system with relatively short reaction and heating times because of comparably small temperature drift, the information necessary for scale-up cannot be obtained.

### Continuous Flow Experiments

To overcome the variations in heating rate and reaction temperature, a continuous-flow reaction system was established. It allows a very precise control of temperature and reaction time, two parameters being crucial for the studied dehydration reaction. The high surface-to-volume ratio of a capillary and the rapid heat transfer through the thin and highly heat-conducting steel wall allow not only for a very fast and accurate heating of the reaction solution to the desired temperature but also for a rapid cooling afterward. The reaction time becomes equal to the residence time in the capillary reactor and can be set by adjusting the flow of the reaction solution. Pressurization to increase the reaction temperature beyond the ambient-pressure boiling point is also simple and safe in a capillary system. All in all, reproducibility using this system was found to be largely superior to the batch reactor.

**Figure 6:**
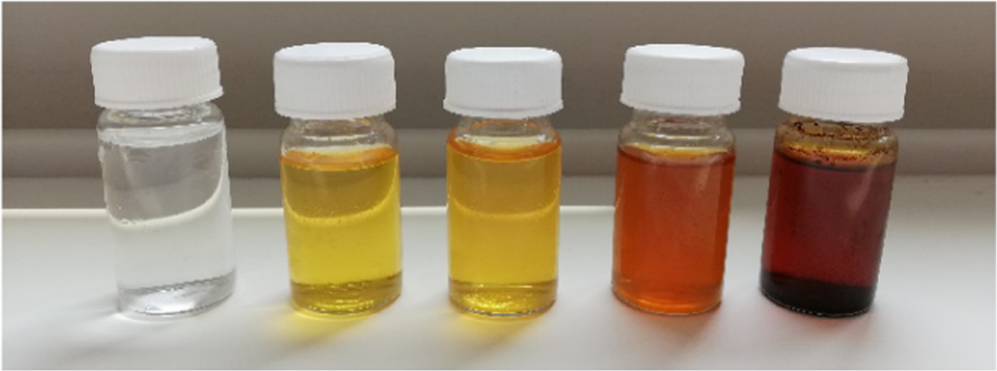
Samples of reaction solutions produced in the microreactor, sorted by rising 5-HMF content.

The main parameters tested with the microreactor were the influence of reaction temperature (160–185 °C), reaction time (0.5–2.5 min), and catalyst concentration (0.1–3% acid) on the yields of 5-HMF and byproducts. The fructose concentration was the same (5%) in all experiments. Typical reaction solutions can be seen in Figure 6. The samples are sorted by 5-HMF content, from 0.1% 5-HMF on the left to 20% 5-HMF on the right. The higher the 5-HMF content, the darker was the reaction solution, indicating that with the yield of the main product also the formation of byproducts increased. As mentioned previously, furanic compounds tend to form extremely potent chromophores.^8^ The coloration of the solution was evidently not derived from the 5-HMF itself, but from these side products, since dissolving pure 5-HMF in similar concentrations does not result in strongly colored solutions.

**Figure 7:**
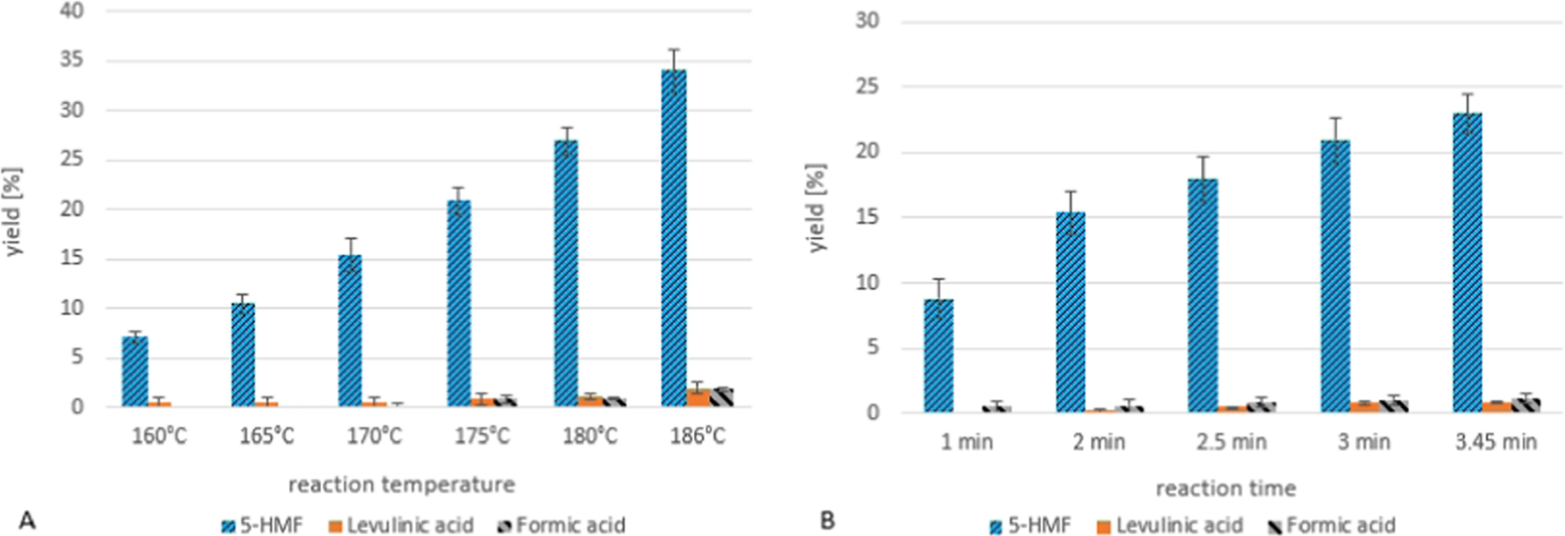
Microreactor setup: (A) influence of reaction temperature on 5-HMF yield, 0.1% H_2_SO_4_, 2 min reaction time, 5% fructose; (*n* = 3); (B) influence of reaction time on 5-HMF yield, 0.1% H_2_SO_4_, 170 °C, 5% fructose (*n* = 3).

**Figure 8:**
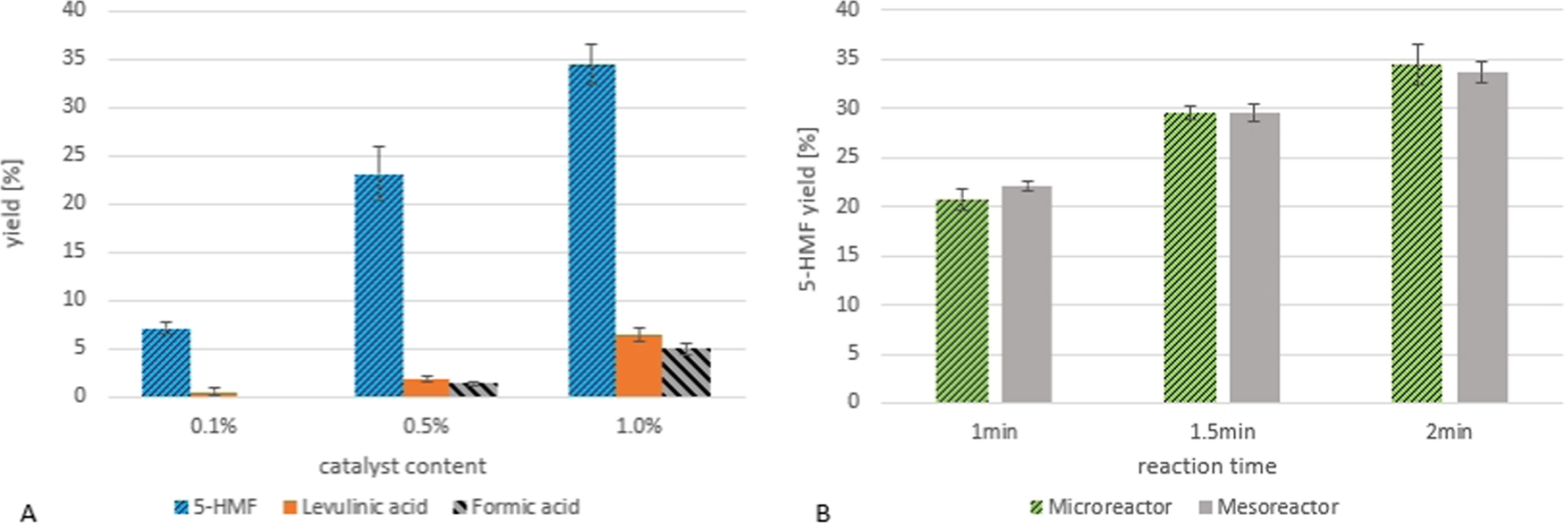
(A) Microreactor setup—influence of catalyst (H_2_SO_4_) concentration on 5-HMF yield; 160 °C; 2 min reaction time; 5% fructose (*n* = 3). (B) Comparison of 5-HMF formation in the microreactor and the mesoreactor at different reaction times; 5% fructose; 1% H_2_SO_4_; 160 °C (*n* = 3).

The influence of the reaction temperature, reaction time, and catalyst content on 5-HMF yields can be seen in Figures 7 and 8. As expected, higher reaction temperatures and higher acid content as well as longer reaction times led to higher 5-HMF yields (Figures 7 and 8A). Shorter reaction times at higher temperatures were preferable due to the higher flow velocity at shorter reaction times, which effectively prevented clogging at a 5% fructose concentration (less byproduct formation).

The system was optimized by stepwise adjustment of the reaction conditions to higher temperatures, higher catalyst concentration, and longer reaction times. When the reaction conditions became too severe (temperature above 180 °C), clogging was observed. After this stepwise optimization, the best yield obtained with the microreactor system was 49% 5-HMF with 8% byproduct formation (formic acid/levulinic acid) at 1% catalyst content, 5% fructose solution, 0.6 min reaction time, and 175 °C reaction temperature. Compared to the literature, this may be classified as a satisfying yield for an aqueous 5-HMF production system. Muranaka et al.^19^ achieved a yield of 60% 5-HMF in their system, but at a lower fructose content of only 1%, which allows the use of more severe conditions without running into clogging issues. Tuercke et al.^18^ achieved a slightly higher yield of 54%, but with a HCl catalyst, which was shown to be a more effective catalyst for this reaction, but was not used in this work due to the reasons discussed above.

Unfortunately, the microreactor setup is limited due to clogging issues. Even at quite low fructose concentrations of 5–10%, fouling and clogging can become a serious problem. The more harsh the reaction conditions became (e.g. acid content >1%, reaction temperature >185 °C), the more side products formed and the more relevant clogging problems became: higher 5-HMF concentrations in the reaction solution cause more polymerization reactions.

Another factor to be considered is the starting fructose concentration. At higher fructose concentrations, lower 5-HMF yields were obtained under otherwise similar reaction conditions. An increase in fructose concentration from 5 to 10% caused an 8% decrease in 5-HMF yield. As mentioned above, the main reason for the decrease in yield is increased byproduct formation. The reaction conditions need to be altered when higher fructose concentrations are to be used. To achieve higher yields, lower reaction times at higher temperatures can be applied. Due to the higher flow velocity and shorter residence time, less byproducts are formed and higher yields are possible without clogging.

The reaction kinetics for the dehydratization of glucose or fructose in a water–H_2_SO_4_ system were modeled by Guo et al. very recently.^28^ The reaction orders for all of the reactions starting from either 5-HMF or the sugars were found to lie in the range of 0.88–1.38. A dependence of first-order reaction on the reactant was assumed for all subreactions. At 135 °C, they found an intrinsic rate constant of *k* = 0.6072 ± 0.0754 L/mol*min with an activation energy of 133 ± 5 kJ/mol for the conversion of fructose to HMF. A steeper increase in the HMF yield than in the side-product yields of levulinic acid and formic acid can be explained by a slightly higher impact of the reaction temperature on the reaction rate of the dehydration reaction of fructose than the rehydration reaction of 5-HMF. The slightly higher yields of formic acid can be explained by the direct decomposition of the sugar to form humins and formic acid. The lower formic acid yield at high catalyst contents might be a result of the incorporation of formic acid into humin structures. Lower yields from glucose can also be explained by looking at the reaction kinetics. The reaction from glucose has a higher activation energy (156 kJ/mol), and the kinetic constants are 2 orders of magnitude lower.

For the direct binder production, a higher starting content of fructose is desirable. Typical solid contents of, for instance, urea-formaldehyde binders range between 63 and 66%.^29^ Although other reactants will be added to the produced precursor solution, the reaction solution will need to be concentrated to serve as a suitable basis for a binder, which has a negative impact on the overall sustainability of the process. The higher the fructose content of the starting solution, the less concentration (solvent evaporation) will be needed.

The system needed to be improved also with regard to the clogging issue. Therefore, a mesoreactor with a bigger inner capillary diameter (2 mm instead of 1 mm) was built to allow the use of more concentrated fructose solutions without running into clogging problems. In comparison to the microreactor, the HMF yields obtained in the mesoreactor are basically the same, which can be seen in Figure 8B.

**Figure 9:**
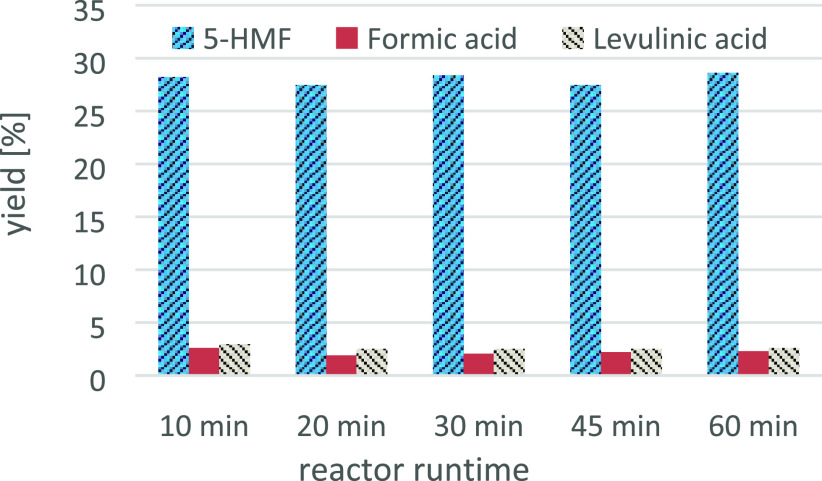
Long-term stability of the mesoreactor, samples taken at different active runtimes; 10% fructose; 1% H_2_SO_4_; 162.5 °C, 55 s reaction time.

The reproducibility is not negatively affected. On the contrary, the mesoreactor system is more stable, although clogging might still occur at fructose concentrations >10%. It usually starts to become eminent between 45 and 60 min of active production time. Figure 9 demonstrates the long-term stability of the system. Several samples were collected during a 1 h run of the reaction system, showing a very constant reaction output over this period of time. The produced reaction solutions are stable and can be stored at room temperature for at least 7 days without a significant change in the composition.

### Utilization of the Produced Solutions in Resin Synthesis

**Figure 10:**
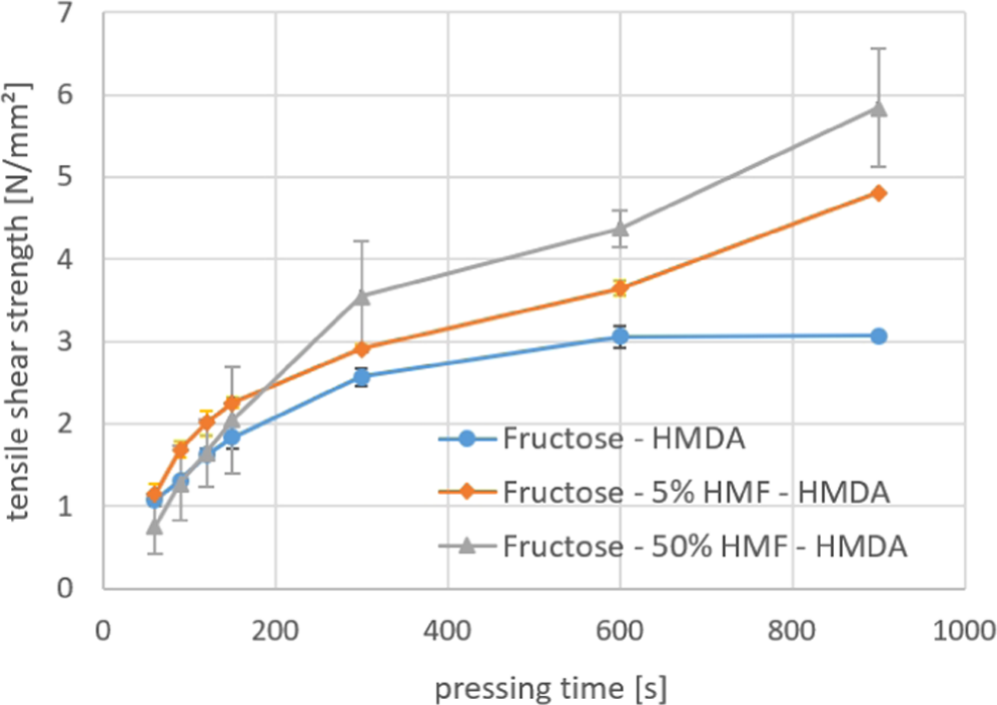
Bond strength development of fructose—5-HMF—hexamethylenediamine (HMDA) resins at 120 °C press temperature.

As mentioned above, the solutions produced in the continuous reaction system are used in the development of new adhesive systems. Figure 10 shows the effect of the substitution of 5 and 50 mol % of fructose by 5-HMF in an amine-fructose adhesive system. It can be clearly seen that the addition of 5-HMF improves the bonding performance of the resin.

More detailed information on the resin synthesis and testing procedure will be published in a following publication. It has to be noted that benchmark urea-formaldehyde resins harden faster than these resin systems at the moment. Therefore, these resin systems must be further improved and developed, to reach industrial requirements.

## Conclusions

A 5-HMF yield of 49% was achieved in the acid-catalyzed dehydratization of fructose in a microreactor system with H_2_SO_4_ as the catalyst (1% catalyst; 5% fructose; 0.6 min reaction time; 175 °C). The long-run stability was improved by switching to a reaction system with a bigger inner diameter (2 mm instead of 1 mm) of the capillary used in the reaction zone. The flow reaction system was found to perform better compared to the tested batch reaction systems, due to a better temperature and reaction time control and thus higher reproducibility.

Further improvements in the design of the mesoreactor as currently studied would involve the transfer of the backpressure regulator to a position between reaction and cooling zone. This should lead to less clogging because less precipitates will pass through the valve. As for temperature control, the rapid heat transfer in this system is very relevant for the reaction. A preheating step may be useful, so the set reaction temperature can be reached even faster. A temperature gradient might lead to lower yields, since already produced product would enter the zone with the desired reaction temperature and is therefore prone to side-product formation for a longer time. In the cooling zone, on the other hand, a temperature gradient or more controlled temperature decrease could lead to less precipitation and therefore may improve the overall system.

In accordance with our incentives, the produced 5-HMF-rich carbohydrate solutions can be used directly for resin applications. The use of organic solvent as well as additional separation and filtration steps were avoided and the usually unwanted humin-type byproducts are employed as contributors to the desired resin binder efficiency. This makes the developed system not only technically feasible but also economically viable for larger-scale production of alternative, bio-based binders.

First, fructose—5-HMF—amine resins were produced, and it could be shown that the bonding performance is improved by 5-HMF addition.

## Supporting Information Available

Typical ^1^H NMR spectrum (reaction solution) (PDF)

## Abbreviations Used

HMF: hydroxymethylfurfural
FDCA: furandicarboxylic acid
DOE: design of experiment
HPLC: high-performance liquid chromatography
AOX: adsorbable organic halogens
NMR: nuclear magnetic resonance
HMDA: hexamethylenediamine
