# Supplementary material for: Sulfuric Acid-Catalyzed Dehydratization of Carbohydrates for the Production of Adhesive Precursors
Source: ACS Omega. 2021 Jun 15;6(25):16641–8. doi: 10.1021/acsomega.1c02075 (PMC8246703; doi:10.1021/acsomega.1c02075)
Supplement: Supplementary file 1 — ao1c02075_si_001.pdf [file ao1c02075_si_001.pdf]

Supplementary information: Sulfuric acid catalyzed dehydratisation of carbohydrates for production of adhesive precursors

Wilfried Sailer-Kronlachner[* † ‡], Catherine Thoma[† ‡], Stefan Böhmdorfer[§], Markus Bacher[§], Johannes Konnerth[‡], Thomas Rosenau[§], Antje Potthast[§], Pia Solt[†] and Hendrikus W.G. van Herwijnen[†].

† Wood K plus – Competence Center of Wood Composites and Wood Chemistry, Kompetenzzentrum Holz GmbH, Altenberger Str.69, A-4040 Linz, Austria

‡ Institute of Wood Technology and Renewable Materials, Department of Material Science and Process Engineering, University of Natural Resources and Life Sciences, Vienna (BOKU), Konrad-Lorenz Str. 24, A-3430 Tulln, Austria

§ Institute of Chemistry of Renewable Resources, Department of Chemistry, University of Natural Resources and Life Sciences, Vienna (BOKU), Konrad Lorenz-Straße 24/I, A-3430 Tulln, Austria

* corresponding author, email: w.sailer-kronlachner@wood-kplus.at, w.sailer@boku.ac.at

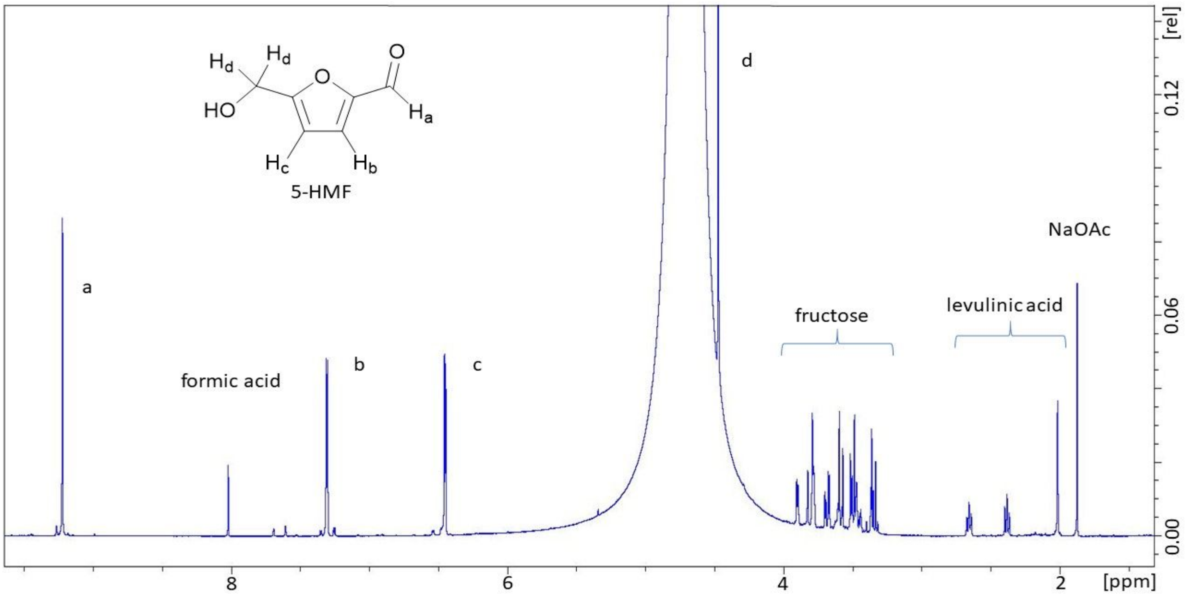

Figure S1: ^{1}H NMR spectrum of a typical reaction solution; CHO peak of 5-HMF at 9.2 ppm (a); formic acid peak at 8 ppm; furan peaks at 7.3 and 6.4 ppm (b,c); fructose peaks between 3.2 and 4 ppm; levulinic acid peaks at 2.6, 2.3 and 2 ppm; NaOAc peak at 1.87 (internal standard)
